# 3,4-diaminopyridine treatment for Lambert-Eaton myasthenic syndrome in adults: a meta-analysis of randomized controlled trials

**DOI:** 10.1186/s12883-021-02405-3

**Published:** 2021-09-25

**Authors:** Na Zhang, Daojun Hong, Taohui Ouyang, Wei Meng, Jingwei Huang, Meihua Li, Tao Hong

**Affiliations:** 1 Department of Neurology, the First Affiliated Hospital of Nanchang University, Jiangxi, China; 2 Department of Neurosurgery, the First Affiliated Hospital of Nanchang University, No.17, Yongwai Street, Jiangxi Province 330006 Nanchang, China

**Keywords:** 3,4-diaminopyridine, Lambert-Eaton myasthenic syndrome, Quantitative myasthenia gravis, Muscle

## Abstract

**Background:**

Lambert-Eaton myasthenic syndrome (LEMS) is a rare autoimmune disorder of neuromuscular transmission. The objective was to examine the efficacy and safety of 3,4-diaminopyridine (3,4-DAP) in patients with LEMS.

**Methods:**

We searched several databases to identify relevant studies, including PubMed, EMBASE, Web of Science, MEDLINE, Cochrane Neuromuscular Disease Group Specialized Register and the Cochrane Central Register of Controlled Trials(CENTRAL). The primary outcome, quantitative myasthenia gravis (QMG) score and the secondary outcome, compound muscle action potentials (CMAP) amplitude were pooled by meta-analysis.

**Results:**

Six randomised controlled trials (RCTs) involving 115 patients with LEMS were included. QMG score showed a significant decrease (improvement) of 2.76 points (95 % CI, -4.08 to -1.45, *p* < 0.001) after treatment with 3, 4-DAP. Moreover, the overall mean CMAP amplitude improved significantly in LEMS patients with 3, 4-DAP treatment, compared with placebo treatment (mean difference 1.34 mV, 95 % CI, 0.98 to 1.70, *p* < 0.001). The overall assessment of all included trials showed a low risk of bias and low heterogeneity.

**Conclusions:**

The pooled results of RCTs demonsrated with moderate to high evidence that 3,4-DAP has a significant effect on LEMS treatment, with improvements in muscle strength score and CMAP amplitude.

## Background

Lambert-Eaton myasthenic syndrome (LEMS) is a rare autoimmune neuromuscular junction dysfunction resulting from antibodies that are generated against voltage-gated calcium channels (VGCC) on presynaptic nerve terminals, thereby suppressing the release of neurotransmitters such as acetylcholine [1–3]. The onset age of LEMS patients mainly ranges from 20 to 50 years [4], although cases in childhood and infancy have been reported [5, 6]. It is estimated that about 60 % of LEMS patients are tumor-related [7]. Among LEMS patients, small-cell lung cancer (SCLC) is the most common type, but other types of tumors, including non-small cell lung cancer, mixed lung cancer, thymoma, and prostate cancer, have also been reported [7, 8]. Based on the prevalence of SCLC, the prevalence of LEMS is estimated to be 1 in 100,000 in the United States [4]. LEMS is characterized by limb girdle muscle weakness, easy fatigability, absence of deep tendon reflexes with post-tetanic potentiation, and autonomic alterations, such as dry mouth and erectile dysfunction. Activities related to daily functions, such as rising from a chair, climbing stairs, and self-care management, are also involved [9]. It has been extensively documented in biopsied intercostal muscles that reducing the quantitative release of ACh plays a vital role in the pathophysiology of LEMS [10].

LEMS is initially diagnosed on the basis of typical clinical characteristics, including the classic triad of proximal muscle weakness, autonomic nerve dysfunction and decreased tendon reflexes [11]. A confirmation diagnosis of LEMS also requires a combination of specific VGCC antibody detection and characteristic electrophysiological results. The detection of positive VGCC antibody provides reliable evidence for the diagnosis of LEMS. VGCC-antibody-positivity has been observed in 100 % of LEMS patients with SCLC and 90 % of LEMS cases without potential malignancy [12]. Lambert and Eaton [13] first proposed the classic electrophysiological triad of LEMS, including low resting compound muscle action potentials (CMAP) amplitude, decreased response of low-frequency repetitive nerve stimulation, and increased response after high frequency stimulation or short exercise.

Over the past few decades, many symptomatic drug treatments have been tried, including guanidine, pyridazine, 4-aminopyridine and 3,4-diaminopyridine (3,4-DAP, amifampridine), the last of which has proven to be the most effective [14]. With the exception of 3,4-DAP, these drugs have not been studied in clinical trials, but only in small case series. In December 2009, 3,4-DAP was approved for the first time in Europe and was subsequently recommended as a first-line treatment for LEMS in 2010 [15]. Amifampridine phosphate (a salt form of 3,4-DAP) was found to be more stable than the base form, as it can be stored at room temperature [16].

In recent years, the efficacy of 3, 4-DAP in the treatment of LEMS has been widely discussed, but the results of the available studies remain uncertain. No reliable conclusions have been drawn and it is unclear whether the potential advantages outweigh the disadvantages. Therefore, we performed a meta-analysis of randomized controlled trials (RCTs) to evaluate the efficacy and safety of 3,4-DAP in the treatment of LEMS in adults.

## Methods

### Literature search

**Fig. 1:**
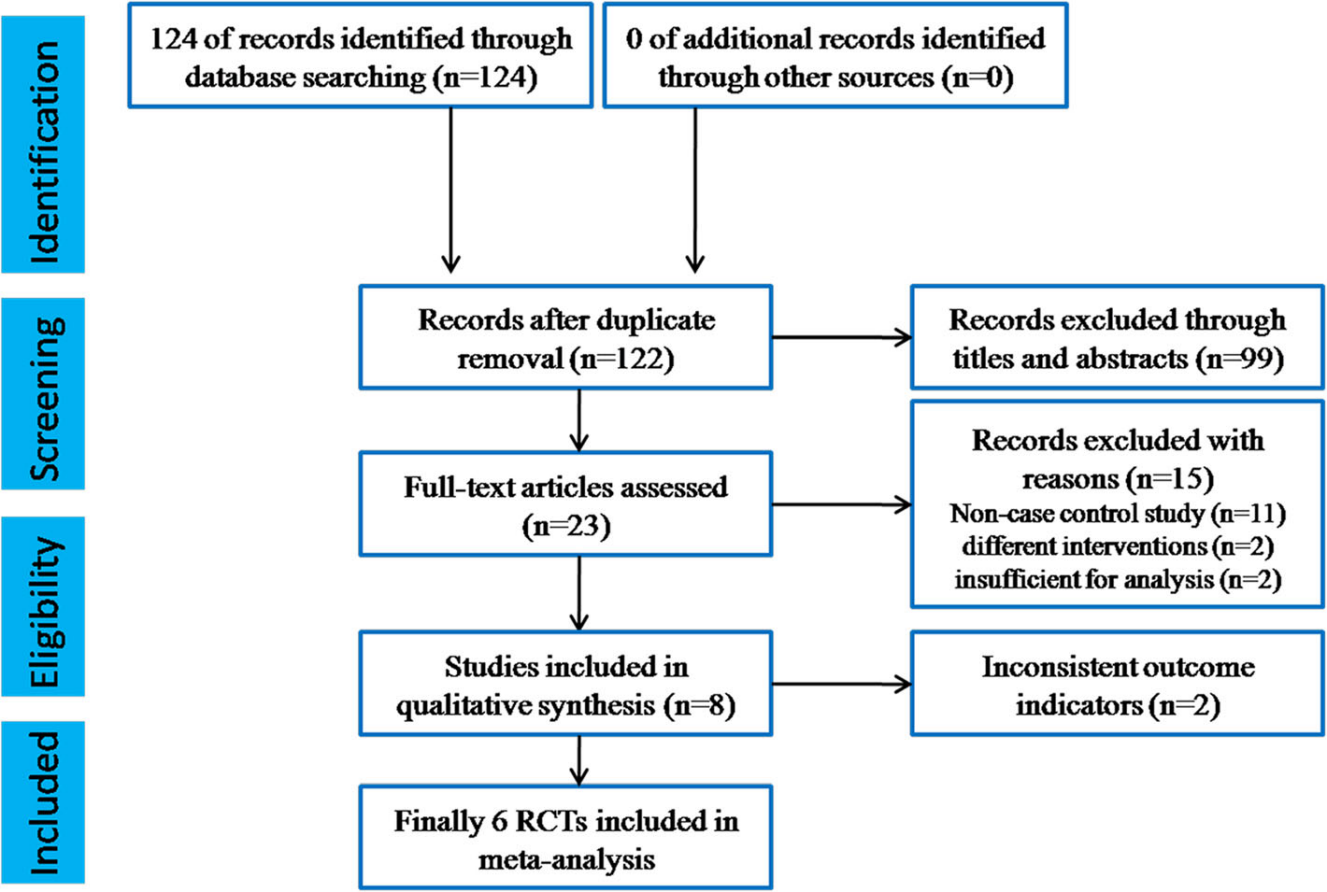
PRISMA flow diagram of the search and select process

A comprehensive search of electronic databases including the Cochrane Neuromuscular Disease Group Specialized Register, the Cochrane Central Register of Controlled Trials (CENTRAL), Pubmed, Web of Science, MEDLINE and EMBASE was performed for all years up to January 2020. The following terms were used to search for pertinent studies: ‘Lambert-Eaton (myasthenic syndrome)’ or ‘Eaton-Lambert’ or ‘LEMS’ combined with ‘3, 4-Diaminopyridine’ or ‘3,4 Diaminopyridine’ or ‘Firdapse’ or ‘Amifampridine Phosphate’ or ‘3,4-DAP’. Bibliographies of the randomized trial reports were consulted and the study authors were contacted to determine other published or unpublished data. By searching these databases, a total of 122 articles were retrieved and 99 articles were deleted by title and abstract. Of the remaining 23 articles, 15 articles were deleted for reasons including no case-control studys, different interventions or insufficient data. Of the remaining 8 studies, two were also deleted because they did not use uniform indicators to evaluate the results. In the end, six articles were included in this meta-analysis. The search and select process is shown in Fig. 1.

### Inclusion criteria

All randomized or quasi-randomized controlled trials of adult patients diagnosed with LEMS, with or without SCLC, and who received 3, 4-DAP treatment were included. The primary outcome indicator was change in muscle strength score (quantitative myasthenia gravis, QMG), or limb muscle strength measured by myometry. The secondary outcome indicator was change in average CMAP amplitude at rest.

### Data extraction

Two reviewers (D.H. and W.M.) independently examined all titles and abstracts retrieved from the literature search of various databases, and evaluated the full text of all potentially related studies. Each reviewer independently assessed the study eligibility and disagreements were resolved by group discussion. Two authors (J.H. and M.L.) independently performed the data extraction. Baseline data were collected using standardized tables, including author, year of publication, number of cases, patient age, sex, presence of cancer, and outcome measures (mean, standard deviation). Whenever possible, insufficient data was obtained from the study authors.

### Quality assessment

**Fig. 2:**
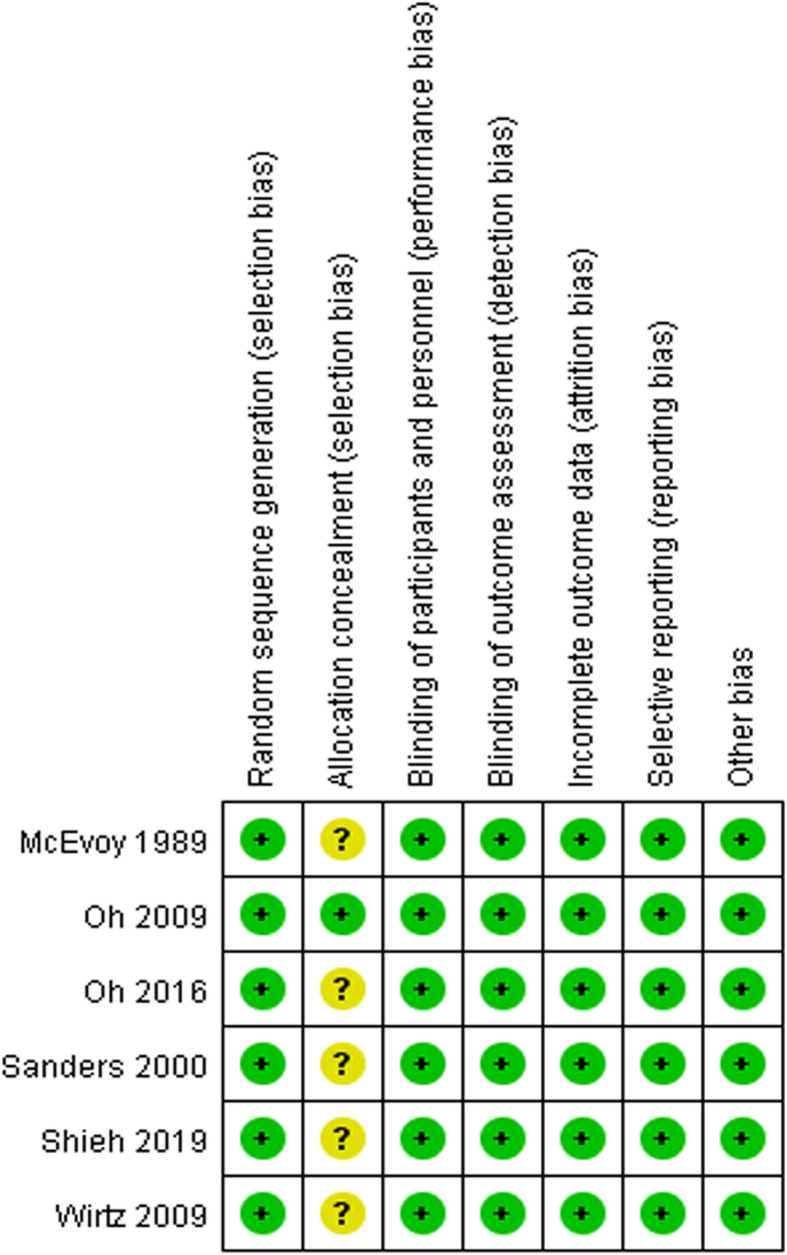
Risk of bias summary: Methodologic quality graph showing review authors’ judgments for each methodologic quality item presented as percentages across all included studies

The methodological quality of each included article was independently assessed by two authors using the Cochrane Collaboration’s tool to determine the risk of bias (Fig. 2). The risk of bias process includes sequence generation, blinding, allocation concealment, selective reporting, processing of incomplete results data, or any other form of bias [17]. The items were rated as “Yes”, “No” or “Unclear” per the established Cochrane scale, with “Yes” representing a low risk of bias, and “No” representing a high risk of bias. ‘Unclear’ was used when there was inappropriate information to make a judgment, or when the project was not relevant to the research. The review authors reached agreement by consensus.

### Sensitivity analysis

Sensitivity analysis was performed on the basis of methodological quality, testing for heterogeneity of results, and adjusting confidence limits as appropriate.

### Statistical analysis

Due to cross-over studies included in this meta analysis, we pooled data with the generic inverse variance (GIV) method, which uses the mean differences (MD) and standard error (SE) of the mean for the diference between treatment and control. Wherever possible, published SE was used;When this was not available, we estimated SE with published P-values or raw data obtained by the authors.MD and 95 % CIs were output to perform the test of combined statistics. All P values were 2-sided, with *P* < 0.05 indicating statistical significance. The heterogeneity of these studies was tested by inconcistency index (I^2^) statistics. An I^2^ > 50 % indicated the existence of substantial heterogeneity, in which case a random effects model was adopted, otherwise, a fixed effects model was used for a pooled analysis [18]. All the statistical analysis was conducted with RevMan (version 5.3, the Cochrane Collaboration, London).

## Results

### Description of studies

The results of the literature search found 8 RCTs that used 3, 4-DAP to treat LEMS. However, in two of these RCTs, 3TUG was used to evaluate the outcome of treatment without the use of QMG or CMAP, therefore the two RCTs had to be excluded as they did not meet the inclusion criteria. Finally, only six RCTs were eligible for inclusion in this meta-analysis. The six eligible RCTs included 115 patients with LEMS treated with oral or intrevenous 3,4-DAP or placebo, without any healthy participants.The characteristics of all included studies are shown in Table 1.

**Table 1:** Characteristics of individual trials included in the meta-analysis

Author	Year	Country	Study type	Study design	Cases	Age (year)	Female (%)	Cancer (%)	Treatment	QMG	CMAP
McEvoy	1989	USA	RCT	Cross-over	12	34–75	8(66.7 %)	7(58.3 %)	3,4-DAP	N/A	CMAP
Sanders	2000	USA	RCT	Parallel	26	41–68	15(57.7 %)	10(38.5 %)	3,4-DAP	QMG	CMAP
Oh	2009	USA	RCT	Cross-over	6	25–75	1(16.7)	2(33.3 %)	3,4-DAP	QMG	CMAP
Wirtz	2009	Netherlands	RCT	Cross-over	9	33–73	4(44.4 %)	N/A	3,4-DAP	N/A	CMAP
Oh	2016	USA	RCT	Parallel	36	21–88	23(63.9 %)	6(16.7 %)	3,4-DAP	QMG	CMAP
Shieh	2019	USA	RCT	Parallel	26	31–75	16(61.5 %)	6(23.1 %)	3,4-DAP	QMG	N/A

*N/A* without data, *QMG* quantitative myasthenia gravis, *CMAP* compound muscle action potentials

The first RCT was a cross-sectional trial of 12 patients with LEMS, which compared the efficacy of the maximum oral dose of 3, 4-DAP (100 mg/ day) with the placebo for 6 days, using muscle strength score and CMAP at days 3 and 6 [19].

The second RCT, a parallel group design, compared an oral dose of 3, 4-DAP (60 mg/day) for 12 participants with placebo for 14 participants. QMG muscle strength score and CMAP were performed on days 5 and 6 [20].

The third RCT was a double-blind, cross-over study of 6 participants. Three patients in the first group received an initial dose of 15 mg per day, that was gradually increased up to a daily dose of 80 mg at the end of the 8-day course.The second group increased their daily intake of 30 mg to 75 mg during the 3-day study because of time constraints. QMG score and CMAP were recorded as the outcomes [21].

The fourth RCT was a double blind, double dummy, cross-over study of 9 participants. It compared intravenous 3,4-DAP against placebo, intravenous pyridostigmine, and a combination infusion of 3,4-DAP and pyridostigmine. Muscle strength and CMAP between 10 and 170 min after infusion were taken as endpoints [14].

The fifth RCT was a double-blind, parallel study. It compared 3,4-DAP for 16 patients with placebo for 20 participants. In part 1, patients received a total dose of 3,4-DAP 15–80 mg/day in 3–4 subdoses, with a maximum single dose of 20 mg. Patients received a dose of at least 30 mg/day to enter the second part of the study.QMG score and CMAP were performed on day 14 [22].

The sixth RCT was a double-blind, parallel study. It compared 3, 4-DAP for 13 patients with placebo for 13 participants. These 13 patients received 3, 4-DAP (30–80 mg/d, 3 or 4 times daily). QMG score was recorded on day 4 [23].

### Data synthesis

#### Primary outcome measure: the score on a muscle strength scale

**Fig. 3:**
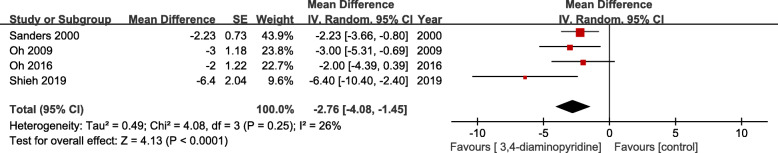
Forest plot for comparison of 3,4-diaminopyridine treatment versus placebo, change in mean QMG scores with generalised inverse variance model (assumed *r* = 0.5 for within-patient treatment effects in cross-over trials)

The six included RCTs used a muscle strength score or myometric limb measurement as an outcome indicator, and reported significant improvements in muscle strength score, or myometric limb measurement after treatment. However, a meta-analysis of all the studies was impossible on account of the obvious differences in the primary outcome of these studies. QMG score was used as the primary outcome to assess muscle strength in four of the RCTs [20–23]. The muscle strength scoring system used by McEvoy et al. [19] and the isometric muscle strength scoring system used by Wirtz et al. [14] were different from the QMG scoring system. The muscle strength scoring systems from McEvoy [19] or Wirtz [14] could not be converted into an equivalent QMG score. Therefore, we only compared the overall therapeutic effects of the four RCTs that reported a QMG score by observing the change in QMG score from baseline to 3, 4-DAP or placebo treatment. As these four RCTs included one cross-over study and three parallel studies, it is necessary to conduct GIV analysis. A GIV analysis of the four RCTs showed that QMG scores decreased (improved) by 2.76 points (95 % CI, − 4.08 to -1.45 points) after treatment with 3,4-DAP (Fig. 3).

#### Secondary outcome measure: changes in the amplitude of resting CMAP

**Fig. 4:**
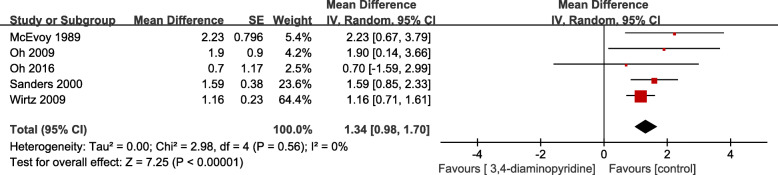
Forest plot for comparison of 3,4-diaminopyridine treatment versus placebo, change in mean CMAP amplitude with generalised inverse variance model (assumed *r* = 0.5 for within-patient treatment effects in cross-over trials)

Changes in resting CMAP amplitude after 3,4-DAP or placebo treatment were recorded in five of the six trials [14, 19–22]. Moreover, all five trials showed significant improvements in CMAP after administration of 3, 4-DAP compared to placebo. As three cross-over studies and two parallel studies were included in the five trials, we conducted a GIV analysis. A meta-analysis of the secondary outcome CMAP showed that the overall CMAP amplitude increased significantly in LEMS patients with 3, 4-DAP treatment, compared with placebo treatment. The overall mean improvement in CMAP of the GIV analysis was 1.34 mV (95 % CI, 0.98 to 1.70), favouring the 3, 4-DAP treatment (Fig. 4). All trials assessed CMAPs at one specific time point during their trial, with only the study of McEvoy and colleagues [19] providing a 3-month follow up of results.

### Publication bias

**Fig. 5:**
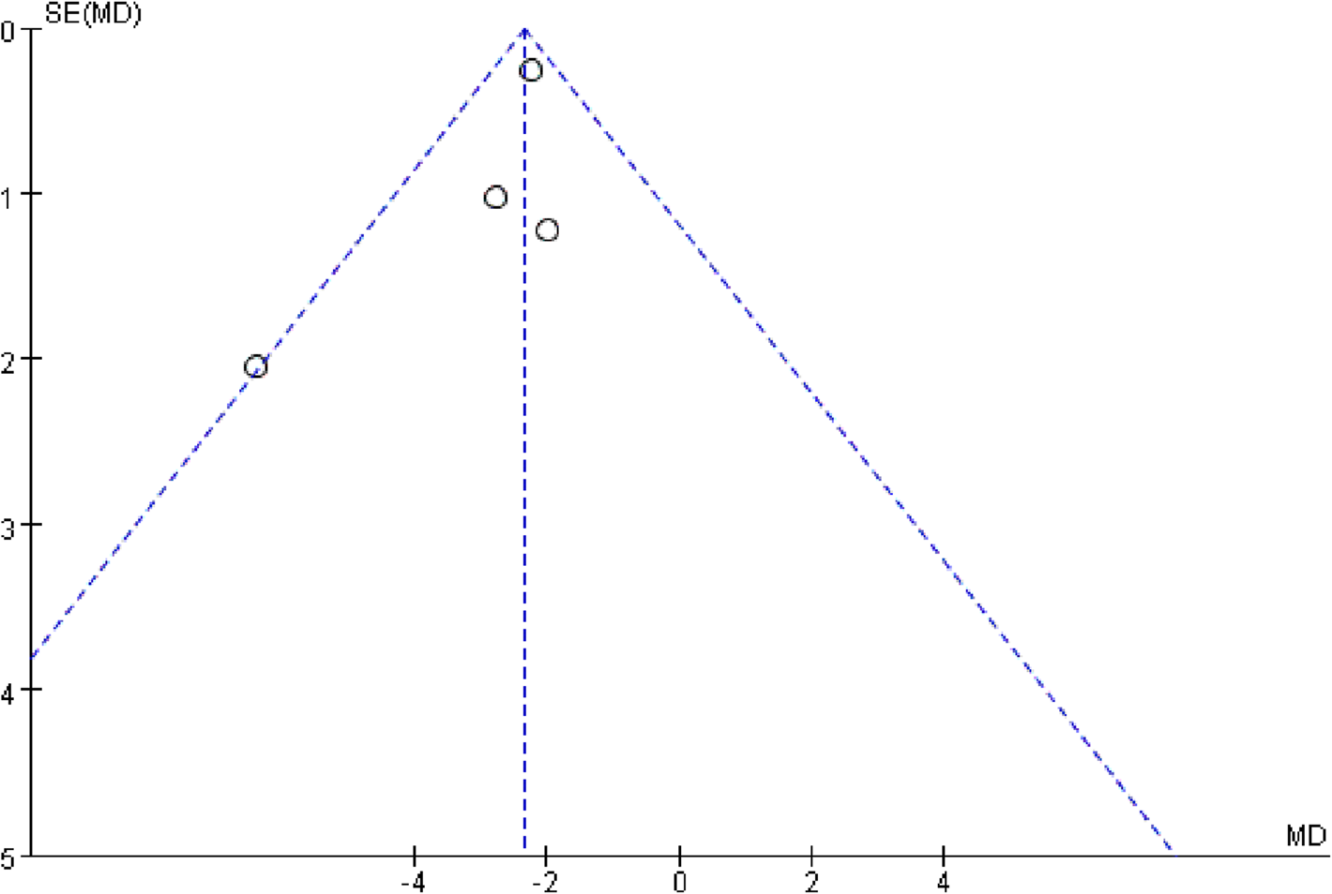
Funnel plot for within study QMG differences between 3,4-diaminopyridine treatment and placebo groups for each trial

**Fig. 6:**
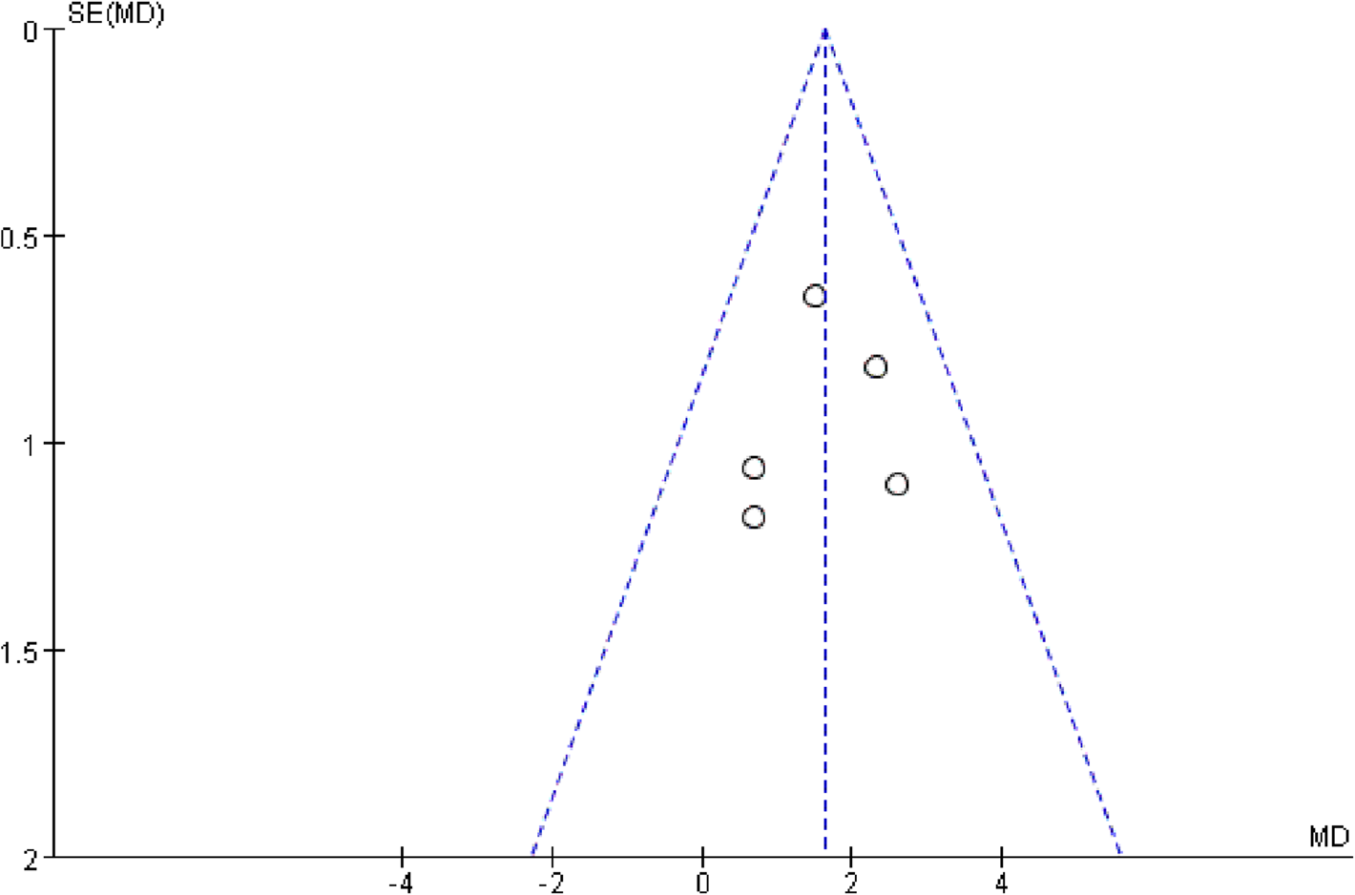
Funnel plot for within study CMAP differences between 3,4-diaminopyridine treatment and placebo groups for each trial

In this meta-analysis, an evaluation of funnel plots showed that the two outcomes QMG and CMAP were basically symmetrical, indicating that the risk of publication bias was small. The funnel plots of the primary outcome QMG and the secondary outcome CMAP are shown in Figs. 5 and 6, respectively. In addition, in order to further accurately assess publication bias, we also performed Begg’s test using StatsDirect statistical software (Version 14.0, StatsDirect Ltd, Cheshire, England). The results of Begg’s test for QMG (*P* = 0.329 [> 0.05]) and CMAP (*P* = 0.760 [> 0.05]) indicated the absence of publication bias.

### Heterogeneity analysis

The heterogeneity tests for both QMG and CMAP showed I^2^ < 50 % (I^2^ = 26 % for QMG and I^2^ = 0 % for CMAP). These results indicate a lack of heterogeneity in these groups and can be considered as coming from a homogeneous group. Thus, combined statistics could be used in fixed effects models.

## Discussion

### Efficacy of 3, 4-DAP treatment for LEMS

In LEMS, 3,4-DAP blocks VGCCs, prolongs the action potential depolarization of motor nerve endings, and increases the opening time of VGCC [24]. This process leads to an increase in presynaptic calcium influx and an improvement in acetylcholine release, manifested as enhancement in muscle function.

In 1989, McEvoy and colleagues studied the effect of oral 3, 4-DAP on 12 LEMS patients, 7 of whom were cancer patients, in a double-blind RCT [19]. This trial showed distinct improvements in neurological dysfunction scores, isometric muscle strength tests, limb strength measurements, autonomic function, and CMAP amplitude after oral 3,4-DAP of up to 100 mg compared to placebo. Oh and colleagues published a cross-over RCT of oral 3,4-DAP of up to 80 mg daily, which indicated a significant effect over the placebo in patients with LEMS [21]. Similarly, another randomized, parallel group trial of 26 patients demonstrated significant enhancement of both QMG score and CMAP amplitude with an oral dosage of 60 mg of 3,4-DAP daily [20].Similar results of intravenous administration of 3,4-DAP were also reported by Wirtz and colleagues [14].

All primary outcome indicators of isometric muscle strength [14], neurological disability score [19] and QMG score [20–23] showed significant improvements after the administration of oral or intravenous 3,4-DAP. We conducted a meta-analysis of QMG score according to the results provided in the studies by Sanders et al.[20], Oh, Claussenet al., Oh, Shcherbakova et al. [21, 22], and Shieh et al. [23]. The QMG rating system, with a score from 0 to 39, is a physician-rated assessment, including assessments of speech, swallowing, external ocular muscles, facial muscle strength, and all limb muscles. The QMG score is a quantitative evaluation, in which a lower score indicates better muscle function [25]. The current meta-analysis showed a significant overall benefit (a decrease of 2.76 points, 95 % CI, -4.08to-1.45) in QMG for LEMS patients with 3, 4-DAP compared to placebo. According to Barohn et al. [26], only a QMG change of more than 2.6 indicates a clinically significant improvement on the basis of 5 myasthenia gravis patients and 4 controls. If Barohn’s criterion is used, this meta-analysis confirmed a significant clinical improvement using 3, 4-DAP, whereas the study by Sanders and colleagues [20] did not show the similar results. As Sanders pointed out, it may be due to the fact that bulbar, ocular, and distal limb items of the QMG score are less common in LEMS than in myasthenia gravis. However, studies by both Oh et al. and Shieh et al. demonstrated that QMG score is an effective method to assess clinical enhancement in muscle strength, provided the QMG scoring system is followed [21–23]. Similarly,Keogh M et al. [27] and Maddison P et al. [28, 29] concurred that the QMG score should still remain the preferred outcome indicator of muscle strength in future LEMS treatment trials. The use of uniform primary outcome measurements and data from further tests will help to describe more specific effects of 3, 4-DAP treatment. We also recommend that in keeping with previous studies, it is appropriate to continue to evaluate the effect of 3,4-DAP treatment by performing a QMG assessment 3–4 days after the initiation of treatment.

Sanders et al. [20] reported that in a RCT of 26 participants with LEMS, the median resting CMAP amplitude improved by 1.3 mV (+ 64 %) in 12 cases receiving 3, 4-DAP 60 mg daily, compared with a decrease of 0.1 mV(-3 %) in the placebo group (*P* < 0.001). This meta-analysis also showed an average improvement in the secondary outcome measure, with a resting CMAP amplitude of 1.34 mV (95 % CI, 0.98 to 1.70). Therefore, the change of mean amplitude of CMAP appears to be a repeatable and objective secondary outcome for any LEMS treatment trial. The authors of this study suggest that since the duration of action of 3, 4-DAP is relatively short, the duration of action should be recorded 3 to 6 h after taking 3, 4-DAP, and recommend that future studies record the CMAP assessment time related to the dose of the drug.

Results from the six RCTs of 3, 4-DAP in the treatment of LEMS showed significant efficacy, consistent with earlier reports of beneficial efficacy. The application of 3, 4-DAP reflects the current practice of first-line symptomatic treatment for patients with LEMS.

### Adverse events

Adverse events associated with 3, 4-DAP treatment reported from the included trials [14, 19–23] included temporary perioral tingling and digital paraesthesiae, back pain, headache, and epigastric discomfort. In addition, Wirtz et al [14] described one participant with cellulitis after 3,4-DAP infusion and McEvoy et al. reported a participant who suffered from epilepsy while taking 100 mg of 3.4-dap daily [19]. Of these included trials, there were no other major adverse events.

### Limitations

Although these RCTs and the pooled results showed a significant improvement in the treatment of LEMS with 3,4-DAP, the number of RCTs studied was relatively small, thus limiting the total number of cases in this meta-analysis. In addition, not all RCTs used the same primary outcome (QMG) and secondary outcome (CMAP) measures. Finally, the follow-up time for these RCTs was limited. In future studies, large sample sizes, consistent outcome measures, and long-term follow-up RCTs are needed to further confirm the therapeutic efficacy and safety of 3,4-DAP on LEMS.

## Conclusions

The pooled results of randomized controlled trials demonsrated with moderate to high evidence that 3,4-DAP has a significant effect on LEMS treatment, with improvements in muscle strength score and CMAP amplitude.

## Data Availability

The datasets supporting the conclusions of this article are included within the article.

## Abbreviations

LEMS: Lambert-Eaton myasthenic syndrome
3,4-DAP: 3,4-diaminopyridine
CMAP: Compound muscle action potentials
QMG: Quantitative myasthenia gravis
RCT: Randomised controlled trial
MD: Mean difference
CI: Confidence intervals
SCLC: Small-cell lung cancer
VGCC: Voltage-gated calcium channel

## References

[1] Bekircan-Kurt CE, Derle Ciftci E, Kurne AT, Anlar B (2015). Voltage gated calcium channel antibody-related neurological diseases. World J Clin Cases.

[2] Yamakage M, Namiki A (2002). Calcium channels–basic aspects of their structure, function and gene encoding; anesthetic action on the channels–a review. Can J Anaesth.

[3] Mantegazza R, Meisel A, Sieb JP, Le Masson G, Desnuelle C, Essing M (2015). The European LEMS registry: baseline demographics and treatment approaches. Neurol Ther.

[4] Sanders DB (2003). Lambert-eaton myasthenic syndrome: diagnosis and treatment. Ann N Y Acad Sci.

[5] Tsao CY, Mendell JR, Friemer ML, Kissel JT (2002). Lambert-Eaton myasthenic syndrome in children. J Child Neurol.

[6] Portaro S, Parisi D, Polizzi A, Ruggieri M, Andreetta F, Bernasconi P, Toscano A, Rodolico C (2014). Long-term follow-up in infantile-onset lambert-eaton myasthenic syndrome. J Child Neurol.

[7] Wirtz PW, Smallegange TM, Wintzen AR, Verschuuren JJ (2002). Differences in clinical features between the Lambert-Eaton myasthenic syndrome with and without cancer: an analysis of 227 published cases. Clin Neurol Neurosurg.

[8] Titulaer MJ, Verschuuren JJ (2008). Lambert-Eaton myasthenic syndrome: tumor versus nontumor forms. Ann N Y Acad Sci.

[9] Harms L, Sieb JP, Williams AE, Graham R, Shlaen R, Claus V, Pfiffner C (2012). Long-term disease history, clinical symptoms, health status, and healthcare utilization in patients suffering from Lambert Eaton myasthenic syndrome: results of a patient interview survey in Germany. J Med Econ.

[10] Newsom-Davis J (2004). Lambert-Eaton myasthenic syndrome. Rev Neurol.

[11] Titulaer MJ, Lang B, Verschuuren JJ (2011). Lambert-Eaton myasthenic syndrome: from clinical characteristics to therapeutic strategies. Lancet Neurol.

[12] Motomura M, Lang B, Johnston I, Palace J, Vincent A, Newsom-Davis J (1997). Incidence of serum anti-P/O-type and anti-N-type calcium channel autoantibodies in the Lambert-Eaton myasthenic syndrome. J Neurol Sci.

[13] Eaton LM, Lambert EH (1957). Electromyography and electric stimulation of nerves in diseases of motor unit; observations on myasthenic syndrome associated with malignant tumors. J Am Med Assoc.

[14] Wirtz PW, Verschuuren JJ, van Dijk JG, de Kam ML, Schoemaker RC, van Hasselt JG (2009). Efficacy of 3,4-diaminopyridine and pyridostigmine in the treatment of Lambert-Eaton myasthenic syndrome: a randomized, double-blind, placebo-controlled, crossover study. Clin Pharmacol Ther.

[15] Kesner VG, Oh SJ, Dimachkie MM, Barohn RJ (2018). Lambert-Eaton Myasthenic Syndrome. Neurol Clin.

[16] Raust JA, Goulay-Dufay S, Le Hoang MD, Pradeau D, Guyon F, Do B (2007). Stability studies of ionised and non-ionised 3,4-diaminopyridine: hypothesis of degradation pathways and chemical structure of degradation products. J Pharm Biomed Anal.

[17] van Tulder M, Furlan A, Bombardier C, Bouter L (2003). Updated method guidelines for systematic reviews in the cochrane collaboration back review group. Spine.

[18] Higgins JP, Thompson SG, Deeks JJ, Altman DG (2003). Measuring inconsistency in meta-analyses. BMJ.

[19] McEvoy KM, Windebank AJ, Daube JR, Low PA (1989). 3,4-Diaminopyridine in the treatment of Lambert-Eaton myasthenic syndrome. N Engl J Med.

[20] Sanders DB, Massey JM, Sanders LL, Edwards LJ (2000). A randomized trial of 3,4-diaminopyridine in Lambert-Eaton myasthenic syndrome. Neurology.

[21] Oh SJ, Claussen GG, Hatanaka Y, Morgan MB (2009). 3,4-Diaminopyridine is more effective than placebo in a randomized, double-blind, cross-over drug study in LEMS. Muscle Nerve.

[22] Oh SJ, Shcherbakova N, Kostera-Pruszczyk A, Alsharabati M, Dimachkie M, Blanco JM (2016). Amifampridine phosphate (Firdapse((R))) is effective and safe in a phase 3 clinical trial in LEMS. Muscle Nerve.

[23] Shieh P, Sharma K, Kohrman B, Oh SJ (2019). Amifampridine Phosphate (Firdapse) Is Effective in a Confirmatory Phase 3 Clinical Trial in LEMS. J Clin Neuromuscul Dis.

[24] Molgo J, Lundh H, Thesleff S (1980). Potency of 3,4-diaminopyridine and 4-aminopyridine on mammalian neuromuscular transmission and the effect of pH changes. Eur J Pharmacol.

[25] Jaretzki A, Barohn RJ, Ernstoff RM, Kaminski HJ, Keesey JC, Penn AS, Sanders DB (2000). Myasthenia gravis: recommendations for clinical research standards. Task Force of the Medical Scientific Advisory Board of the Myasthenia Gravis Foundation of America. Neurology.

[26] Barohn RJ, McIntire D, Herbelin L, Wolfe GI, Nations S, Bryan WW (1998). Reliability testing of the quantitative myasthenia gravis score. Ann N Y Acad Sci.

[27] Keogh M, Sedehizadeh S, Maddison P. Treatment for Lambert-Eaton myasthenic syndrome. Cochrane Database Syst Rev. 2011;2011(2):CD003279. 10.1002/14651858.CD003279.pub3 PMC7003613 21328260

[28] Maddison P, Newsom-Davis J. Treatment for Lambert-Eaton myasthenic syndrome. Cochrane Database Syst Rev. 2005;(2):CD003279. 10.1002/14651858.CD003279.pub2 15846654

[29] Maddison P (2012). Treatment in Lambert-Eaton myasthenic syndrome. Ann N Y Acad Sci.

